# Essential role for Batf3-dependent dendritic cells in regulating CD8 T-cell response during SARS-CoV-2 infection

**DOI:** 10.1371/journal.pone.0294176

**Published:** 2023-12-27

**Authors:** Liat Bar-On, Hani Dekel, Moshe Aftalion, Theodor Chitlaru, Noam Erez

**Affiliations:** 1 Department of Biochemistry and Molecular Genetics, Israel Institute for Biological Research, Ness-Ziona, Israel; 2 Veterinary Center for Preclinical Research, Israel Institute for Biological Research, Ness-Ziona, Israel; 3 Department of Infectious Diseases, Israel Institute for Biological Research, Ness-Ziona, Israel; Lerner Research Institute - Cleveland Clinic, UNITED STATES

## Abstract

SARS-CoV-2 infection elicits robust CD8 T-cell responses, yet the identity of the mechanisms playing dominant roles in initiating the virus-specific CD8 T-cell responses are largely unknown. In the present study, we interrogate the contribution of the cDC1 subset to SARS-CoV-2-specific CD8 T-cell immunity. For this purpose, we used a novel murine line which combines the SARS-CoV-2 susceptible K18-hACE2 transgenic and the Batf3 deficient mice which lack the cDC1 subset. We demonstrate that in the absence of cDC1, viral-specific CD8 T-cell responses were severely impaired both in the draining lymph node as well as in the lungs, during the effector phase of SARS-CoV-2 infection. Furthermore, SARS-CoV-2 specific memory CD8 T-cells in the lungs and spleens were also significantly impacted, whereas humoral responses, as well as CD4 T-cells were not affected. Additionally, we demonstrate that the absence of cDC1 subset, and the consequent impaired CD8 T-cell responses, resulted in significant increase in SARS-CoV-2 viral load in the lungs. The conclusions of the study were further independently corroborated in an additional COVID-19 murine model consisting infection with a mouse-adapted SARS-CoV-2 virus. These results underscore a specific role for Batf3-dependent DC in regulating SARS-CoV-2 specific CD8 T-cell responses and may contribute to future vaccine design and immunization strategies.

## Introduction

The worldwide COVID-19 pandemic, caused by severe acute respiratory syndrome virus type two (SARS-CoV-2), has prompted numerous investigations addressing specific host-pathogen interactions. These studies were enabled by the availability of novel animal models in particular murine which were instrumental to understanding the immune responses elicited by SARS-CoV-2 infection. The K18-hACE2 mouse model expressing the hACE2 transgene is a widely used model for studying SARS-CoV-2 pathogenesis and evaluation of antiviral prophylactics and countermeasures. In spite of its ability to emulate the COVID-19 disease in mice, this model suffers of the limitation of high lethality associated with SARS-CoV-2 neuro-invasion, preventing long-term studies [1–4]. Recently, we and others have documented that a sublethal primary infection of K18:hACE2 mice may be achieved by administration of low virus doses. This mild infection elicited robust SARS-CoV-2-specific cellular and humoral responses [5, 6].

It is well accepted that CD8 T-cells play a prevalent role in protective immunity against viruses of different types [7]. Recent studies have shown that respiratory infection with SARS-CoV-2 virus elicits robust CD8 T-cell response [8–10], yet the mechanisms inducing these CD8 T-cells responses are still elusive. Antigen presentation to CD8 T-cells by antigen presenting cells (APC) can occur by direct presentation of endogenously synthesized antigens or by cross-presentation of exogenously acquired antigens [11, 12]. Conventional dentritic cells (cDCs) are highly specialized antigen-presenting cells, acknowledged as key initiators and regulators of T-cell-mediated immunity [13–17]. A key paradigm in cDC function is their ability to uptake antigens in peripheral tissues, deliver them to draining lymph nodes (dLNs), undergo maturation and upregulate major histocompatibility (MHC) and co-stimulatory molecules required for T-cell priming and activation [18, 19]. Conventional DCs comprise two major functionally distinct subsets, namely cDC1 and cDC2. The lineage cDC1 consists of lymphoid organ-resident CD8^+^ DCs and their tissue-residing and migratory counterparts, characterized by high expression of CD103 (hence, CD103^+^ DCs) [20]. The functional human equivalent of murine cDC1 is the CD141^hi^ DC subset [21]. The development of cDC1 is uniquely dependent on the basic leucine zipper transcription factor ATF-like 3 (Batf3). Thus, mice deficient in Batf3 selectively lack cDC1, without compromising the development of other DC subsets and immune cells [20, 22]. Studies with Batf3 deficient mice (Batf3^-/-^) have demonstrated that the cDC1 subset exhibits unique antigen cross-presenting capabilities and consequently, these cells play critical roles in antiviral and antitumor immunity via the generation of cytotoxic T cell responses [22–26]. Batf3-deficient mice represent a key model for elucidating the *in vivo* functions of cDC1. Indeed, an accumulating wealth of data has demonstrated that cDC1 occupy a central position in regulating various aspects of CD8 T-cell responses to viral infections: Batf3-dependent lung cDC1s are required for transport of viral antigens to the mediastinal lymph node (MLN), where they induce CD8 T-cell immunity against influenza or Sendai virus [20, 26, 27]. Upon challenge with respiratory influenza virus, Batf3^-/-^ mice failed to induce protective immunity, suggesting a protective role for cDC1 in viral infections [26]. It was also shown that cDC1 are required for memory CD8 T-cells following vaccinia virus (VACV) respiratory infection [23]. In some cases, such as CMV infection, the cDC1 subset is essential for CD8 T-cell early acute response while dispensable in later stages [28]. Conversely, CD8 T-cell response to infection with the intestinal Rotavirus (RV) does not appear to require cDC1 involvement [25]. Thus, the involvement of cDC1 appears to vary in scope and intensity amongst various viral pathogens. Whether Batf3-dependent DC modulate antiviral immunity during respiratory SARS-CoV-2 infection remains unknown.

In the present study, we interrogate the contribution of the cDC1 subset to SARS-CoV-2-specific CD8 T-cell immunity. For this purpose, we used a novel murine line obtained by combining the SARS-CoV-2 susceptible K18-hACE2 transgenic mice with the Batf3^-/-^ mice.

## Materials and methods

### Ethics statement

All mice used in this study were maintained according to the guidelines and regulations for animal experiments at the Israel Institute for Biological Research (IIBR). All animal experiments were approved by the IIBR Institutional Animal Care and Use Committee (IACUC) (protocol numbers M-11-22, M-16-21 and M-38-19).

### Cells and viruses

Vero E6 (ATCC® CRL-1586TM), Calu-3 (ATCC HTB-55), HEK293T (ATCC CRL-11268) and HEK293-hACE2 (GenScript M00770) cells were maintained in Dulbecco’s Modified Eagle’s Medium (DMEM) supplemented with 10% fetal bovine serum (FBS), MEM non-essential amino acids, 2 nM L-Glutamine, 100 Units/ml Penicillin, 0.1 mg/ml streptomycin and 12.5 Units/ml Nystatin (Biological Industries, Israel). Cells were cultured at 37°C, 5% CO2 and 95% humidity air atmosphere. SARS-CoV-2 (Germany/BavPat1/2020, GISAID accession EPI_ISL_406862) was kindly provided by Bundeswehr Institute of Microbiology, Munich, Germany and propagated in Vero E6 cells. Mouse Adapted SARS-CoV-2 (MA10 variant, NR-55329) was described previously [29, 30], obtained through BEI Resources, NIAID, NIH and propagated in Calu-3 Cells. All viruses were tittered by plaque assay on Vero E6 cells as described previously [31], aliquoted and stored at −80°C until use.

### Animal experiments

C57BL/6J (JAX 00064), Batf3^-/-^ (JAX 013755) and K18-hACE2 (JAX 034860) were purchased from The Jackson Laboratory (Bar Harbor, ME). Generation of K18-hACE2/Batf3^-/-^ mice was achieved by crossing of Batf3^-/-^ and K18-hACE2 heterozygous mice. The resulting Batf3^+/-^/K18hACE2^+/-^ were crossed again with Batf3^-/-^ mice. The progeny of this cross consisted a Mendelian ratio of ∼25% hACE2/Batf3^-/-^ and ∼25% hACE2/Batf3^+/-^ mice which served as littermate controls (See S1 Fig). To validate the genotyping of pups, we followed the protocol provided by the Jackson Laboratory husbandry for K18-hACE2 (https://www.jax.org/Protocol?stockNumber=034860&protocolID=38276) and Batf3 (https://www.jax.org/Protocol?stockNumber=013755&protocolID=18022).

For infection, virus stocks were diluted in phosphate buffered saline (PBS) supplemented with 2% FBS (Biological Industries, Israel). Animals were anesthetized by intraperitoneal (i.p.) injection of Ketamin and Xylazine (Ketamine 75 mg/kg, Xylazine 7.5 mg/kg in PBS) and were infected by 30μl intranasal (i.n.) instillation of 50pfu (SARS-CoV-2) or 10^5^pfu (MA10) per mouse.

For CD4 T-cell depletion animals were injected i.p with 0.2mg anti CD4 antibody (clone 1.5GK from BioXcell) one day before infection followed by repeated injections every other day until end of experiment. CD4 T-cell depletion was verified by flow cytometry.

### Tissue processing

At designated time points post infection, mice were euthanized by i.p. injection of Pentobarbiton (300mg/kg) and organs were collected. Lungs were cut and digested with 4 mg/ml collagenase D (Roche, Mannheim, Germany) for 1 h at 37°C. The digested lungs were further mechanically processed and filtered to generate single cell suspensions followed by Red Blood Cells lysis (#R7757, Merck). Mediastinal lymph nodes were incubated with filtered 1mg/ml collagenase D for 30 minutes at 37°C. The digested lymph nodes were further mechanically processed and filtered through 70μm cell strainer (#352350, Falcon), to generate single cell suspensions. Spleens were dissociated into single-cell suspensions in GentleMACS C-tubes (Miltenyi Biotec, Bergisch Gladbach, Germany), filtered, separated on 1.084gr/ml Ficoll Paque premium (#17–5446, GE) and washed with medium (RPMI-1640, 10% FCS, 2mM Glutamine, 1mM Sodium Pyruvate, non-essential amino acids, 25mM HEPES, 5x10^-5^M β-mercaptoethanol and Pen/Strep/Nystatin antibiotics, all from Biological Industries, Israel).

### Flow cytometry

All washing steps were done using FACS buffer (PBS + 0.5% FBS + 2nM EDTA). The following mAb clones were used for staining: CD3 (145-2C11), CD8 (53–6.7), CD4 (GK1.5), CD11b (M1/70), MHCII (I-A/I-E), CD11c (N418), CD103 (2E7), CD62L (MEL-14). All antibodies were purchased from Thermo Fisher Scientific or BD Biosciences. H-2K(b) SARS-CoV-2 S539-546 (VNFNFNGL) tetramers were provided by the NIH Tetramer Core Facility. Dead cells were excluded with LIVE/DEAD Aqua Fixable Dead Cell Stain Kit (Invitrogen). For tetramer staining, cells were pretreated with the protein kinase inhibitor Dasatinib (#SML2589, Merck) at 50nM in PBS for 30min at 37°C. Single-cell suspensions were first stained for viability with Live/Dead reagent in PBS for 30 minutes on ice. Next, samples were incubated with anti CD16/CD32 blocking antibody (#16–0161, Thermo Fisher) and stained for extracellular markers using the antibodies listed above, diluted in FACS buffer for 30 minutes. Following staining, cells were washed with FACS buffer twice, fixed with 3% paraformaldehyde for 20 minutes and washed again twice with FACS buffer. Samples were acquired on LSRFortessa (BD Biosciences) and analyzed with FlowJo V.10 software (TreeStar, Ashland, OR).

### ELISpot assay

For detection of IFNγ-secreting cells, 4 × 10^5^ cells from each spleen sample (see above) were plated into 96-well ELISpot plates in duplicate and incubated for 24hr at 37°C in the presence of the following peptides, presenting immunodominant epitopes, at final concentration of 2μg/ml: Spike amino acids 539–546 VNFNFNGL (S539) and amino acids 915–922 VLYENQKL (S915) as MHC class I H-2K(b) restricted epitopes [32, 33] and ORF3 amino acids 266–280 EPIYNEPTTTTSVPL as MHC class II, I-Ab restricted epitope [34]. Visualization of IFNγ-secreting cells was performed using a Mouse IFN-γ single-Color ELISpot kit (Cellular Technology Limited, Biotec, Bonn, Germany) according to the manufacturer’s instructions. Quantification of cytokine-secreting cells was determined with an ImmunoSpot S6 Ultimate reader and analyzed with ImmunoSpot software (Cellular Technology Limited, Bonn, Germany). Antigen-free cells supplemented with medium were used as a negative control.

### Pseudovirus neutralization assay

Pseudoviruses were produced by co-transfection of plasmids encoding a luciferase reporter (pGreenFire1 from SystemBiosciences), lentivirus backbone (psPAX from Addgen), and S genes (Δ19 S-covid-pCMV3, gift from Yossi Shaul, Weizmann Institute of Science, Israel) into HEK293T cells, using Lipofectamine3000 (Nunc #L3000001) according to the manufacturer protocol. 48 hours later the medium was collected, cleared by centrifugation, filtered through a 0.45μm filter and stored at -80°C for future use. One day before pseudovirus neutralization assay, 2x10^4^ hACE2 expressing HEK293 cells were plated in each well of 96well plate (Thermo Fisher#136101). On the day of assay, serial dilutions of heat-inactivated serum were mixed with pseudoviruses, incubated, and then added to hACE2-expressing 293T cells. 24 hours later, cells were lysed and luciferase activity (in relative light units (RLU)) was measured. Per cent neutralization was normalized considering uninfected cells as 100% neutralization and cells infected with only pseudovirus as 0% neutralization. IC_50_ titres were determined using a log (agonist) vs normalized-response (variable slope) nonlinear function using Prism software (GraphPad).

### Determination of the viral load in organs

At specific time points post infection, mice were euthanized and their lungs were removed into PBS. Tissues were processed by homogenizer (ULTRA-TURAX® IKA R104) followed by centrifugation (300g/10min/4°C). The supernatants were collected and kept at -80C until use. To determine virus titer, 10-fold serial dilutions in MEM supplemented with 2% FBS, NEAA and L-glutamine were used to infect VeroE6 monolayers in triplicates in 12-well plates. After 1hr incubation at 37°C an overlay (MEM supplemented with 2% FBS, NEAA and L-glutamine, 0.15% Bicarbonate and 0.4% tragacanth). After 48hr (SARS-CoV-2) or 72hr (MA10) the overlay was aspirated and monolayers were fixed and stained by Cristal Violet solution (0.5% Cristal Violet,20% Ethanol). Plaques were counted and virus titers were calculated.

### Statistical analyses

Statistical analyses were performed using GraphPad software 8.1.1 (La Jolla, CA). All comparisons were made using either Mann-Whitney U test or one-way ANOVA test with Tukey corrected multiple comparisons where p<0.05 = *, p<0.005 = **, p<0.0005 = *** were considered significantly different among groups.

## Results

To probe the involvement of cDC1 in the induction of CD8 T-cell responses following SARS-CoV2 infection, a novel murine line combining susceptibility to SARS-CoV-2 and deficiency of cDC1 cells was generated. For this purpose, Batf3^-/-^ mice (to be referred as Batf3 KO) which lack the cDC1 subset [22] were crossed with the well-established SARS-CoV-2 sensitive transgenic line, expressing the human ACE2 gene (K18-hACE2) [3, 4, 35]. The resulting murine line (see scheme in S1 Fig), to be hereafter referred as hACE2/Batf3KO, was compared to littermate control mice expressing wild-type levels of Batf3 gene (to be referred as hACE2 mice). A complete loss of cDC1 subset (CD103+CD11b-) was confirmed in the lungs of the hACE2/Batf3KO mice at steady-state as well as following SARS-CoV-2 infection (S2 Fig).

We have recently documented that a sublethal infection of K18-hACE2 mice may be achieved upon administration of low SARS-CoV-2 doses. This sublethal infection elicited robust SARS-CoV-2-specific cellular and humoral responses [5]. To determine whether lack of cDC1 affects the susceptibility to respiratory SARS-CoV-2 infection, the above described mice were intranasally inoculated with a low dose (50pfu) of SARS-CoV-2. Following infection, the animals were monitored for disease symptoms and weight loss for 14 days. Both hACE2/Batf3KO and hACE2 control mice exhibited similar minute morbidity as evaluated by weight loss (S3 Fig) with no additional morbidity symptoms.

In order to evaluate the CD8 T-cell response in the presence or absence of cDC1 cells, we conducted a set of experiments where mice were infected with SARS-CoV-2. At different time points post infection mice were sacrificed and organs were collected for further analyses (S4 Fig).

### Impaired induction of SARS-CoV-2-specific CD8 T-cells response in the absence of cDC1

**Fig 1:**
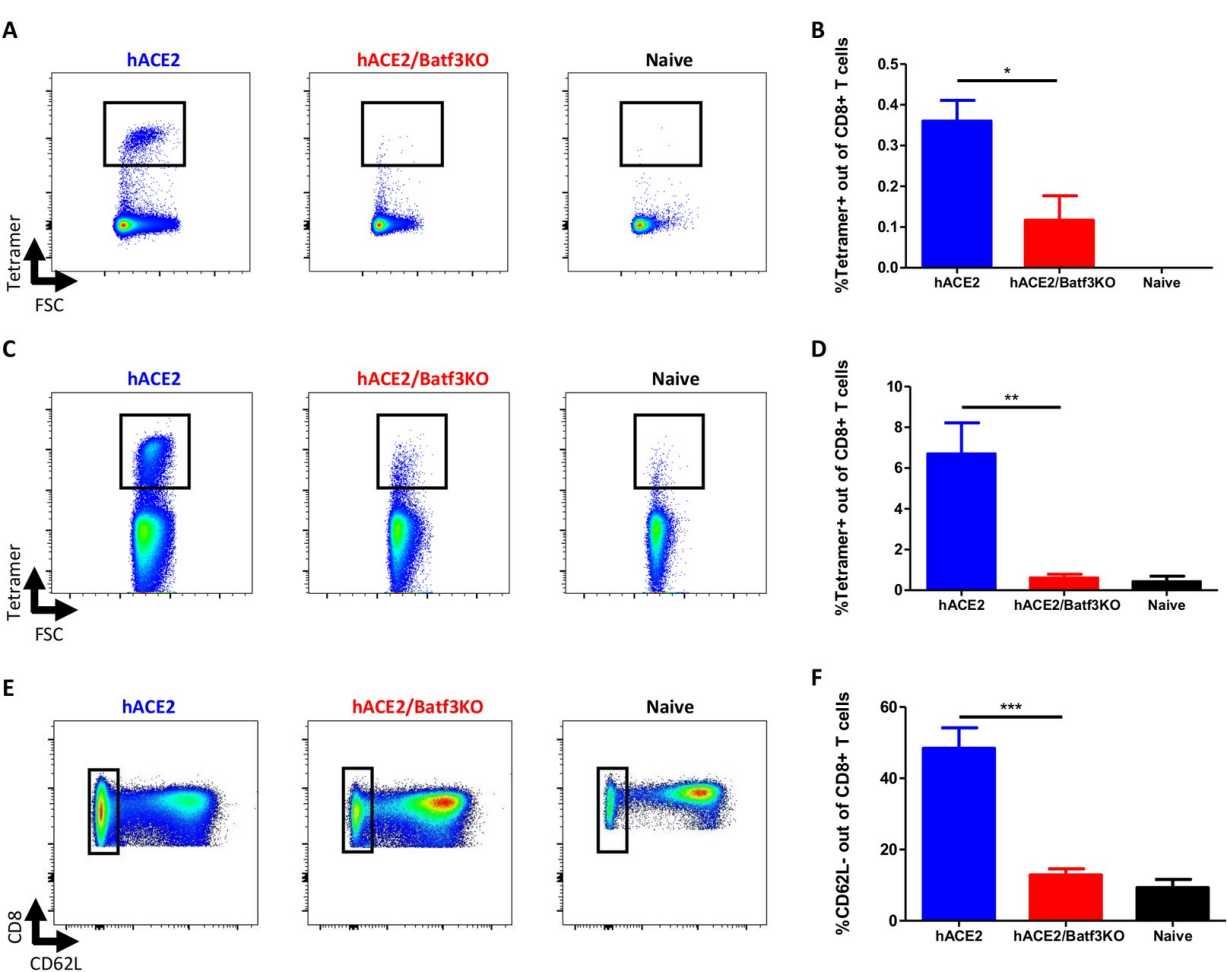
The cDC1 subset is required for priming of CD8 T-cells in the MLN and lungs following SARS-CoV-2 infection. hACE2/Batf3KO (red) and hACE2 (blue) mice were infected i.n with 50pfu of SARS-CoV-2. Results obtained with naïve, non-infected mice, are also depicted (black). At day 8 post infection SARS-CoV-2-specific CD8 T-cells were quantified by tetramer staining in the MLN (A-representative FACS analysis from each group and B-histograms incorporating the individual sets of results obtained for each animal) and the lung (C and D, see explanation for A and B above). Frequencies of effector cells were evaluated by CD62L staining (E and F, see explanation for A and B above). Bars indicate means ± SEM from 3–6 animals per group. Gating strategies for A-B and C-E appear in S5 and S6 Figs, respectively. P values: *P < 0.05, **P < 0.01.

At day 8 post infection, the frequency of SARS-CoV-2-specific CD8 T-cells in the lungs and the draining mediastinal lymph node (MLN) was determined using H2-Kb-S539 epitope MHC-I tetramer staining [34, 36]. While a distinctive tetramer positive CD8 T-cell subpopulation was apparent in the MLN of hACE2 mice (Fig 1A and 1B), a significant reduction (p<0.05) in the frequencies of SARS-CoV-2 specific CD8 T-cells was distinguished in the MLN of cDC1-deficient mice (Fig 1A and 1B). This suggests that cDC1 subset is essential for priming of SARS-CoV-2 specific CD8 T-cells in the draining lymph node. Analysis of SARS-CoV-2 specific CD8 T-cells in the lungs demonstrated a robust accumulation of tetramer positive CD8 T-cells (approx. 7% of total CD8 T-cells) in hACE2 mice. However, in the absence of cDC1, SARS-CoV-2 specific CD8 T-cell accumulation in the lungs was severely compromised (Fig 1C and 1D). This was further substantiated by the observation that the frequencies of activated CD8 T-cells (CD62L-) were also considerably reduced in the lungs of hACE2/Batf3KO mice which were in this respect indistinguishable to the naïve mice (Fig 1E and 1F). In conclusion, these results suggest a critical role of the cDC1 subset in induction of CD8 T-cells immunity during the acute/effector phase of SARS-CoV-2 infection.

### Impaired CD8 memory T-cell response but not humoral response in the absence of cDC1

**Fig 2:**
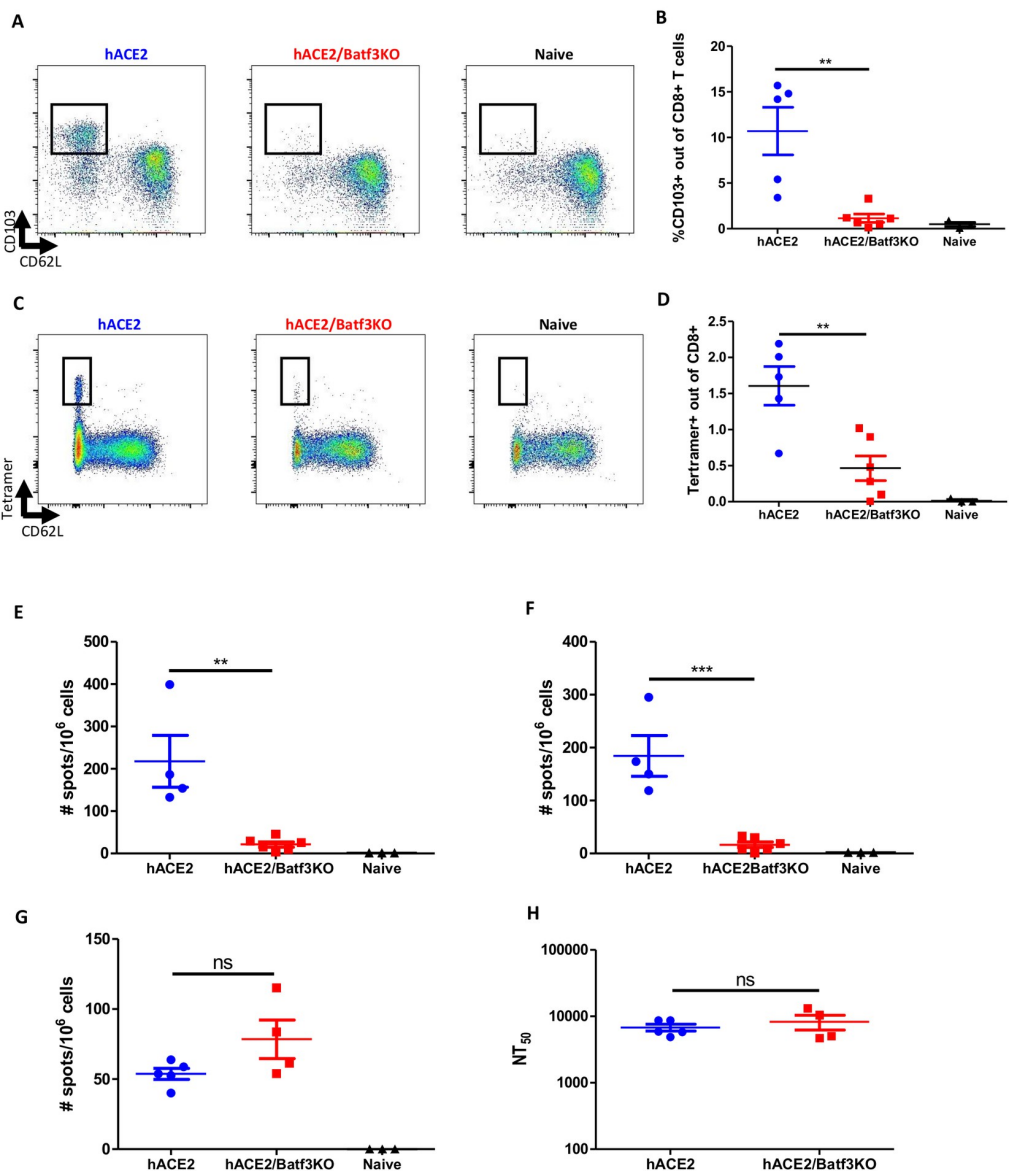
The cDC1 subset is required for induction of CD8 memory T-cells following SARS-CoV-2 infection. hACE2/Batf3KO (red) and hACE2 (blue) mice were infected i.n with 50pfu of SARS-CoV-2. Results obtained with naïve non-infected mice are also depicted (black). 7 weeks post infection the frequencies of mucosal imprinted CD103^+^ memory CD8 T-cells was evaluated (A-representative FACS analysis from each group and B- histograms incorporating the individual sets of results obtained for each animal). Memory CD8 T-cells specific for SARS-CoV-2 in the lung were quantified by tetramer staining (C and D, as above). ELIspot assays were performed using splenocytes from infected animals stimulated with the S539 (E) and S915 (F) peptides. SARS-CoV-2 specific CD4 T-cells were quantified on splenocytes from infected animals using ORF3 peptide (G). Neutralizing antibody titers were determined by pseudovirus neutralization assay (H). Bars indicate means ± SEM from 4–6 animals per group. Gating strategy for A-D appear in S7 Fig. P values: *P < 0.05, **P < 0.01, ***P < 0.001, and ns, not significant.

In order to investigate the role of cDC1 subset in eliciting memory response to SARS-CoV-2, the immune response 7 weeks post SARS-CoV-2 infection in both hACE2/Batf3KO and control hACE2 mice, was analyzed. Data depicted in Fig 2A established that the frequency of mucosal imprinted CD103^+^ memory CD8 T-cells was significantly reduced (p<0.01) in the lungs of cDC1-deficient mice (Fig 2B). Most notably, 7 weeks post infection, the frequency of SARS-CoV-2-specific memory CD8 T-cells (tetramer positive) was substantially reduced in the lungs of hACE2/Batf3KO mice (Fig 2C and 2D). The effect of cDC1 absence on SARS-CoV-2 systemic memory T-cells was assessed also in the spleens by ELISpot assay using two immunodominant SARS-CoV-2-specific CD8 epitopes (namely, S539 and S915) [32]. The data in Fig 2E and 2F demonstrate that 7 weeks post viral infection, high numbers of SARS-CoV-2 specific CD8 T-cells were present in the spleens of control mice. In contrast, the magnitude of the T-cell response to each of these CD8 epitopes was diminished in cDC1-deficient mice (Fig 2E and 2F). Of note, SARS-CoV-2-specific splenic CD4 T-cells responses were equivalent in hACE2 and hACE2/Batf3KO mice (Fig 2G). In line with this observation, similar SARS-CoV-2 neutralizing antibodies titers were measured in both strains of mice (Fig 2H).

### Clearance of SARS-CoV-2 virus in Batf3-deficeint mice

**Fig 3:**
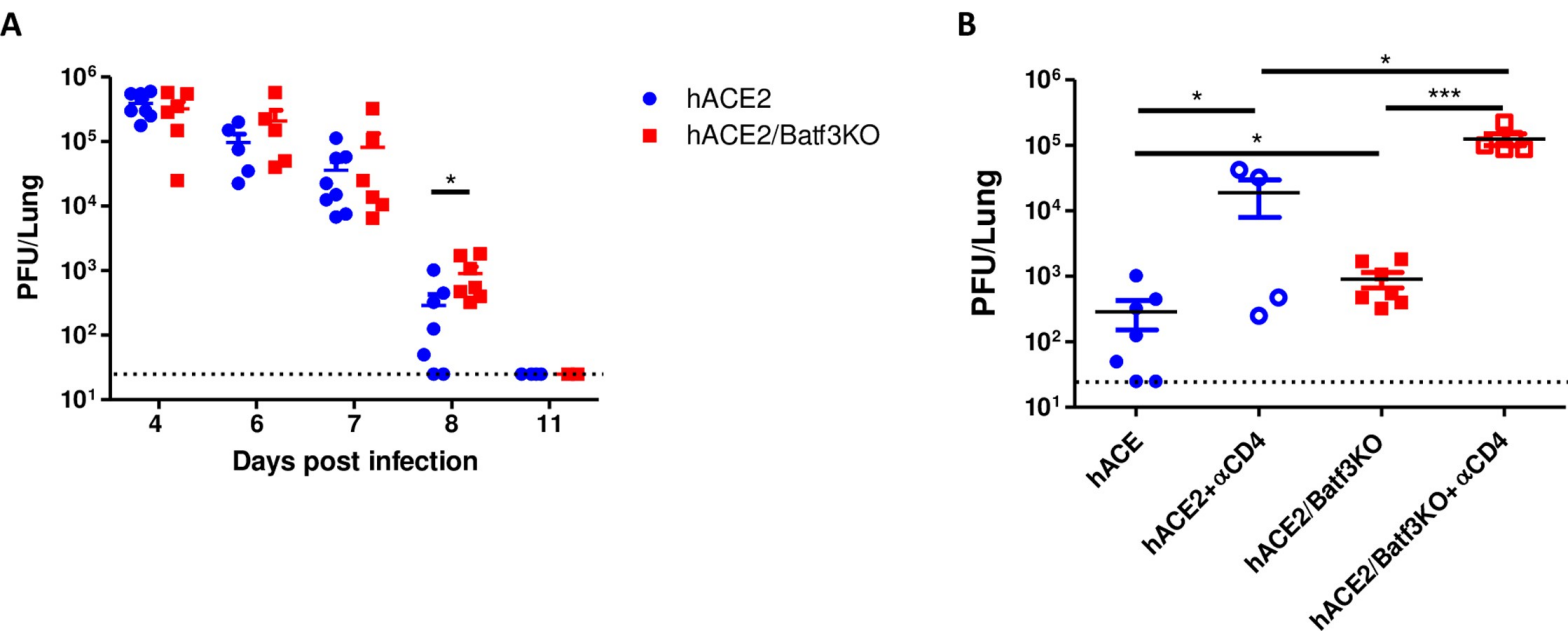
cDC1 cells contribute to SARS-CoV-2 clearance from infected lungs. hACE2/Batf3KO (red) and hACE2 (blue) mice were infected i.n with 50pfu of SARS-CoV-2. At different time points post infection viral loads in the lungs were determined by plaque assay (A). Viral loads in the lungs of hACE2 or hACE2/Batf3KO mice at day 8 post infection following depletion of CD4 T-cells (B). Bars indicate means ± SEM from 4–8 animals per group. P values: *P < 0.05, **P < 0.01, ***P < 0.001, and ns, not significant.

Antiviral CD8 T-cell responses are known to play a major role in mediating the clearance of numerous viruses [7]. Thus, the effect of the impaired CD8 T-cell responses, observed in the absence of cDC1 during SARS-CoV-2 infection, on the clearance of the virus, was addressed. Viral loads in lungs were assessed at several time points post infection in hACE2/Batf3KO and control hACE2 mice. The data in Fig 3A depict a robust virus propagation in infected lungs of both mouse strains at 4 dpi with 50pfu, reaching a viral load of almost 10^6^pfu. These high levels were maintained in both strains in the following 3 days. By day 8, a substantial reduction in viral loads was detected in both experimental groups, yet the titer in Batf3-deficient mice was significantly higher. By day 11 in both groups of infected mice the viral loads declined beneath the limit of detection. These results suggest that SARS-CoV-2 specific CD8 T-cell response contributes, yet unexpectedly is not essential, for the clearance of SARS-CoV-2 from the lungs of infected mice. The observation that cDC1-deficient mice were able to clear the virus, suggested that other mechanisms may have mediated the clearance despite the poor CD8 T-cell response. In order to address the contribution of CD4 T-cell response to viral clearance in the absence of cDC1 subset, and the consequent CD8 T-cell response, we treated hACE2 and hACE2/Batf3KO mice with an anti-CD4 depleting antibody before and throughout SARS-CoV-2 infection and compared the pulmonary viral load at day 8 post infection. Data in Fig 3B clearly shows that depletion of CD4 T-cells (S8 Fig), on the background of impaired CD8 T-cell response, abolished clearance of the virus since viral loads were at day 8 as high as during days 4–7 post infection. This suggests that in the background of impaired CD8 T-cell responses, the involvement of CD4 T-cells in viral clearance is clearly manifested (Fig 3B).

### Confirmation of the essential role of cDC1 subset in induction of CD8 T-cell response, using a mouse adapted SARS-CoV-2 model

**Fig 4:**
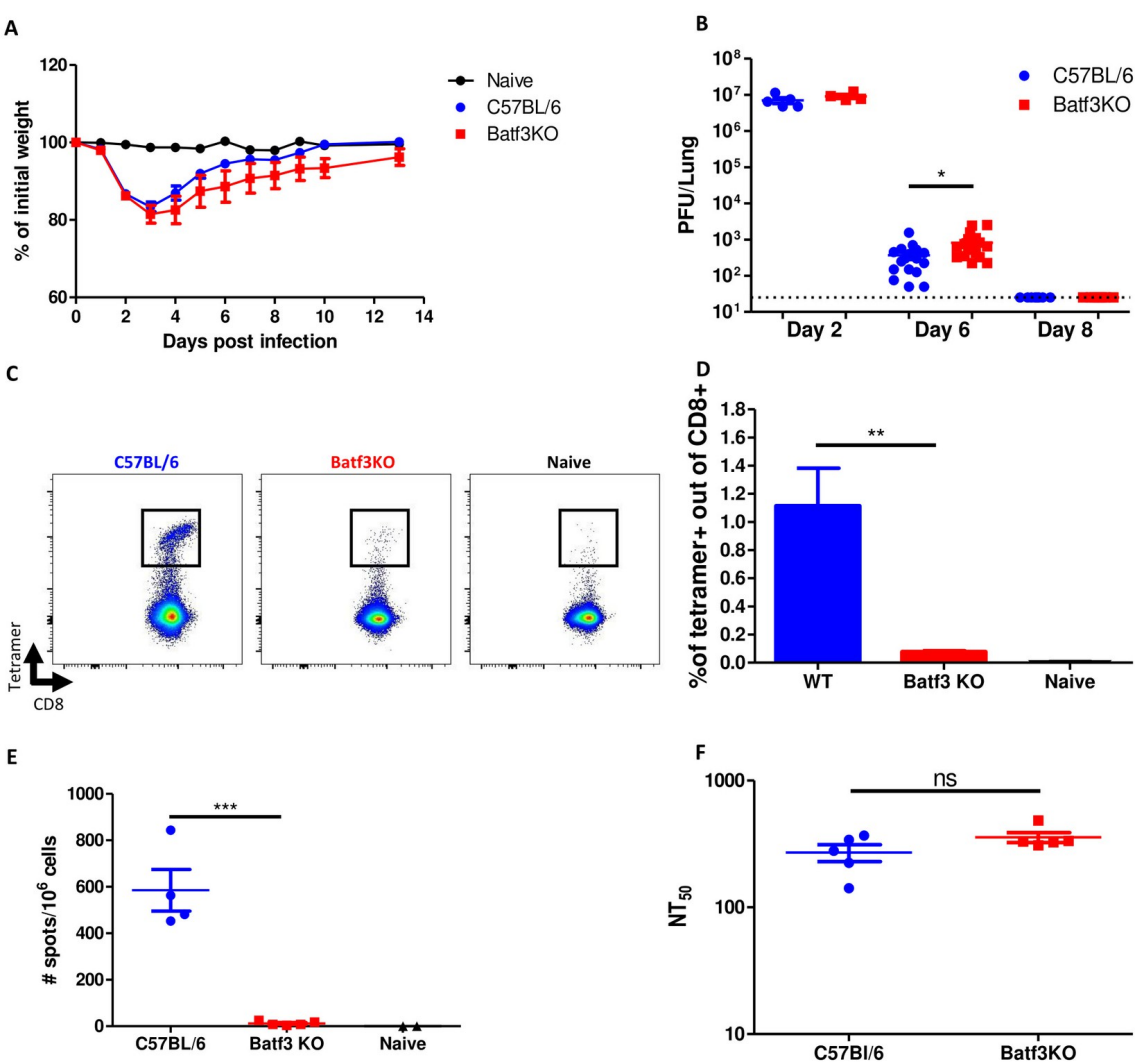
Confirmation of the cDC1 subset requirement in activation of CD8 T-cells using a mouse-adapted SARS-CoV-2. Batf3-/- (red) and C57BL/6 (blue) mice were infected i.n with 10^5^pfu of mouse-adapted SARS-CoV-2 (MA10). Morbidity (weight loss) of infected animals was monitored for 14 days (A). At different time points post infection viral loads in the lungs were determined by plaque assay (B). Five weeks post infection, animals were sacrificed and analyzed for the presence of SARS-CoV-2-specific CD8 T cells by tetramer staining (C and D). ELIspot assays were performed using splenocytes from infected animals stimulated with the S539 peptide (E). Neutralizing antibody titers were determined by pseudovirus neutralization assay (F). Bars indicate means ± SEM from 4–6 animals per group. P values: *P < 0.05, **P < 0.01, ***P < 0.001, and ns, not significant.

The impact of cDC1 on disease progression and the subsequent immune response following intranasal infection, was further corroborated using an additional mouse model for SARS-CoV-2 infection. Accordingly, Batf3 KO and parental C57BL/6 mice (WT) were infected intranasally with the mouse-adapted SARS-CoV-2 virus (MA10) and monitored for weight loss. Both mouse strains exhibited substantial and similar weight loss during the first 3 days post infection, followed by gradual recovery until day 10 post infection (Fig 4A). This observation substantiates the data collected from the previous experiments with hACE2 mice and strengthens the conclusion that the absence of cDC1 cells does not affect the severity of SARS-CoV-2 infection. Consistent with the data gathered from our hACE2 model, significantly higher viral load (p<0.05) was determined in the lungs of cDC1-deficient mice in comparison to WT mice at day 6 post infection. By day 8, the viral loads were undetectable in both strains (Fig 4B). To determine whether in this animal model cDC1-deficincy results in impaired antiviral CD8 T-cell immunity as well, virus-specific CD8 T-cell responses in the lungs and spleens were examined. A substantial population of tetramer positive CD8 T-cells was apparent in the lungs of WT mice, reaching 1% of total CD8 T-cells. In contrast, a significant reduction (p<0.05) in the frequencies of tetramer positive CD8 T-cells was observed in the lungs of cDC1-deficient mice (Fig 4C and 4D). The systemic CD8 T-cell response was evaluated in spleens of infected animals by ELIspot assay. In accordance with the results in the lungs, and with the results from the hACE2 mouse model, documented above, a significant reduced SARS-CoV-2 specific CD8 T-cell population was observed in Batf3 KO in comparison to WT mice (Fig 4E). Neutralizing antibodies titers were comparable in both mouse strains (Fig 4F).

## Discussion

CD8 T-cells fulfil key functions for the control of viral infections. Upon viral infection, antigen presenting cells carry viral antigens to the draining lymph node to activate naive CD4 and CD8 T-cells. The activation of CD8 T-cells in the LN can be carried out by different subsets of DCs. In the present study we addressed the role of a specific DC subset, namely cDC1, in the induction of CD8 T-cells upon infection with SARS-CoV-2, the causative agent of COVID-19. A novel murine line was generated, by genetically combining the well-characterized transgenic K18-hACE2 and the cDC1 deficient Batf3 KO strains. In order to avoid the encephalitic manifestations of the disease occurring in the K18-hACE2 model [4] and to allow for a prolonged disease progression, the mice were infected with a low dose of SARS-CoV-2 (S3 Fig and [5]). The low inoculation dose did not preclude SARS-CoV-2 infection of the lungs and the virus propagated reaching a load of more than 3 orders of magnitude within 4 days of infection. Most importantly, the early and late immune responses following SARS-CoV-2 primary infection could be evaluated.

This report documents that Batf3-dependent DCs were strictly required for the mounting of SARS-CoV-2-specific CD8 T-cell response. The data demonstrated that in the absence of cDC1, priming of SARS-CoV-2 -specific CD8 T-cells is severely reduced, resulting in low frequencies of these cells in the draining lymph nodes as well as their accumulation in the lungs which is almost diminished. The importance of cDC1 cells is manifested also at later phases post infection, indicative of their role in the elicitation of SARS-CoV-2-specific local (lung) and systemic (spleen) memory CD8 T-cells. Of note, although CD8 T-cell responses were severely compromised, induction of memory CD4 T-cells and SARS-CoV-2 neutralizing antibodies titers were not affected in the absence of cDC1 subset. To the best of our knowledge, the present study is the first demonstration of the essentiality of cDC1 subset for induction of CD8 T-cells following SARS-CoV-2 infection. The absence of cDC1 subset and the abrogated CD8 T-cell response did not contribute to further morbidity. This is in contrast to the findings in COVID-19 patients, where a correlation was found between diseases severity and impairment in DCs and CD8 T-cell response (Zhi et al. 2020). This inconsistency could be explained by the limited morbidity in our model and by the long duration of the disease in humans in comparison to murine models.

With regard to the cDC1-dependent activity of SARS-CoV-2 CD8 T-cells, our data implies that CD8 cells contribute substantially to clearance of SARS-CoV-2 as attested by the viral load in the lungs: during the first 7 days post infection, virus loads were similar in both hACE2/Batf3KO and the hACE2 control mice. However, at day 8 post infection, when CD8 T-cell response is apparent in cDC1 competent control mice, cDC1-deficient mice exhibited significantly higher viral loads. These observations are in line with the notion recently reported by Israelow and colleagues [37] suggesting significant role of CD8 T-cells in mediating SARS-CoV-2 clearance (using mice modified to express human ACE2 by AAV transduction). Surprisingly, we have demonstrated that at later stages of infection (day 11) SARS-CoV-2 was cleared from the lungs in both mouse strains, suggesting that SARS-CoV-2-specific CD8 T-cells are not essential for viral clearance. These observations may also indicate that in the absence of a virus-specific CD8 T-cell response, other mechanisms are sufficient to clear pulmonary SARS-CoV-2 during primary infection. This data is in line with studies conducted in rhesus macaques documenting that depletion of CD8 T-cells during primary SARS-CoV-2 infection delayed viral clearance, yet the infection was ultimately resolved by other mechanisms [38]. We demonstrate that in the absence of SARS-CoV-2-specific CD8 T-cell, the significant reduction in viral load observed at day 8 post infection is largely mediated by CD4 T-cells. Consequently, one may presume that CD4 T-cells possess effector mechanisms, yet to be defined, which are instrumental for SARS-CoV-2 clearance. These putative mechanisms are not associated neither with induction of humoral immunity (which typically is detected at later time points) nor with their function as helpers of viral-specific CD8 T-cells.

The use of COVID-19 animal models is often associated with limitations owing to the difficulty in recapitulating the human COVID-19 disease. Therefore, it is important to confirm experimental observations in more than one model. Accordingly, the data collected from the novel K18-hACE2 derived mice was corroborated in an additional murine model employing a mouse-adapted SARS-CoV-2 virus. We show in this model as well, that in the absence of cDC1, CD8 T-cell priming and formation of memory cellular responses are abrogated. These results also correlate with a significant reduction of viral clearance and, in line with the hACE2 model, neutralizing antibody generation were not affected. Hence, both models support the major conclusion that cDC1 cells are instrumental to mount SARS-CoV-2-specific CD8 T-cells response.

In conclusion, we provide an insight to the role of cDC1 subset in the immune response to SARS-COV-2 infection. While CD8 T-cell activation is severely impaired, other arms of the immune response remain intact. This study may contribute to future considerations in design of vaccines and immunization strategies.

## Supporting information

S1 Fig Generation of a novel murine line combining the SARS-CoV-2 sensitive K18-hACE2 transgenic mice with Batf3-/- mice. The resulting hACE2 (blue) and hACE2/Batf3KO (red) littermates, identified by PCR-assisted genotyping of tail DNA samples, were used for infection experiments.
(PDF)

S2 Fig Characterization of lung DC in hACE2 (blue) and hACE2/Batf3KO (red) mice. cDC1 (CD103+CD11b-) and cDC2 (CD103-CD11b+) identification by flow cytometry (A). Quantification of cDC1 and cDC2 in hACE2 and hACE2/Batf3KO mice (B).
(PDF)

S3 Fig Monitoring of weight loss in hACE2 and hACE2/Batf3KO mice following low-dose infection (50pfu) with SARS-CoV-2. (PDF)

S4 Fig Schematic of experimental plans. hACE2/Batf3KO (red) and hACE2 (blue) mice were infected with SARS-CoV-2 by i.n. instillation. Mediastinal lymph nodes and Lungs were collected at days 4, 6, 7, 8 and 11 post infection for further analyses (indicated). 7 weeks post infection spleens, lungs and blood were collected for memory immune response evaluation. Graphical illustration was made with Biorender.com.
(PDF)

S5 Fig Gating strategy for evaluation of SARS-CoV-2 specific CD8 T-cells in the mediastinal lymph node. (PDF)

S6 Fig Gating strategy for evaluation of effector and SARS-CoV-2 specific CD8 T-cells in the lung. (PDF)

S7 Fig Gating strategy for evaluation of memory and SARS-CoV-2 specific CD8 T-cells in the lung. (PDF)

S8 Fig Confirmation of CD4 T-cell depletion. Animals were treated with anti CD4 depleting antibody one day before infection and in every other day post infection. CD4 T-cell depletion was confirmed by flow cytometry.
(PDF)
