# Supplementary figures and images for: Essential role for Batf3-dependent dendritic cells in regulating CD8 T-cell response during SARS-CoV-2 infection
Source: PLoS One. 2023 Dec 27;18(12):e0294176. doi: 10.1371/journal.pone.0294176 (PMC10752548; doi:10.1371/journal.pone.0294176)

S1 Fig

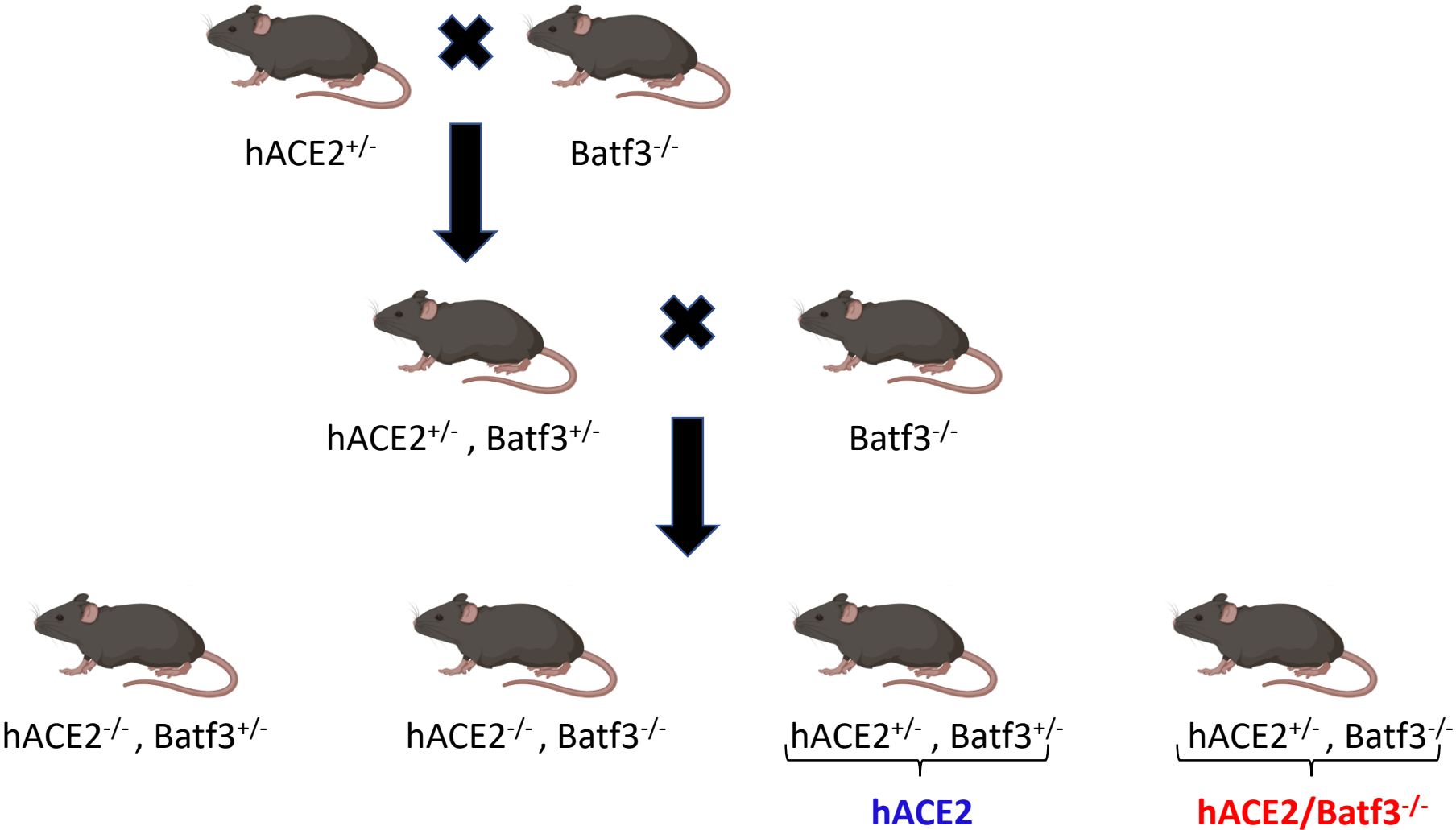

Supplement: S1 Fig — The resulting hACE2 (blue) and hACE2/Batf3KO (red) littermates, identified by PCR-assisted genotyping of tail DNA samples, were used for infection experiments. (PDF) [file pone.0294176.s001.pdf]

S2 Fig

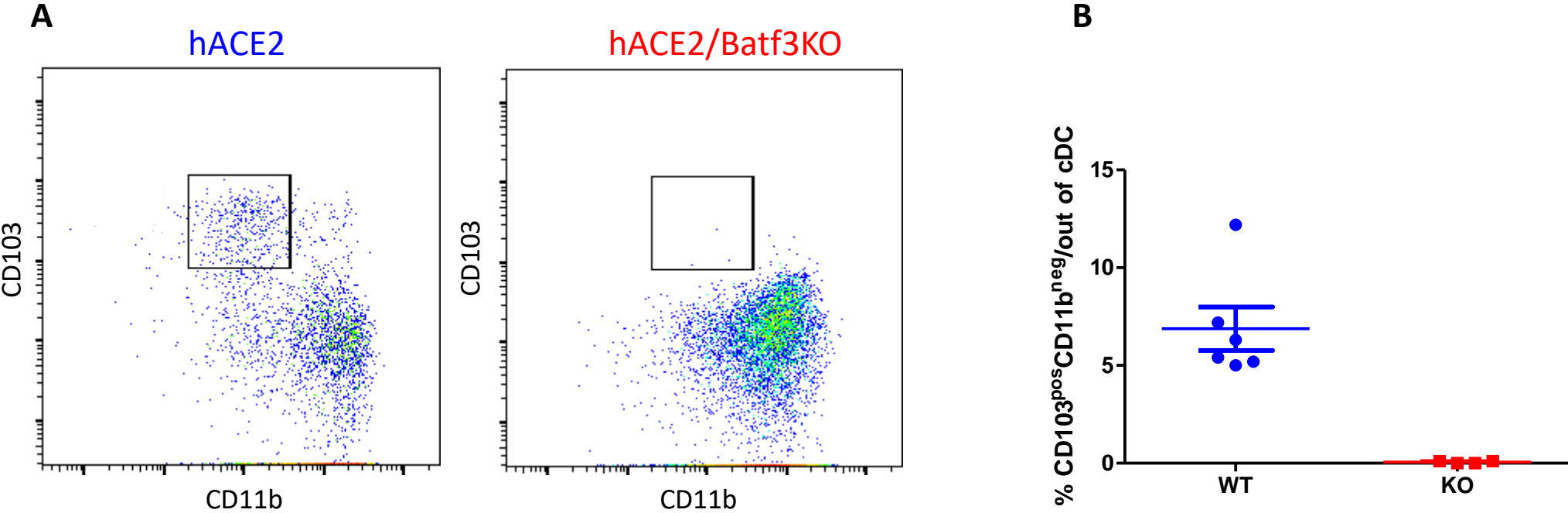

Supplement: S2 Fig — cDC1 (CD103+CD11b-) and cDC2 (CD103-CD11b+) identification by flow cytometry (A). Quantification of cDC1 and cDC2 in hACE2 and hACE2/Batf3KO mice (B). (PDF) [file pone.0294176.s002.pdf]

S3 Fig

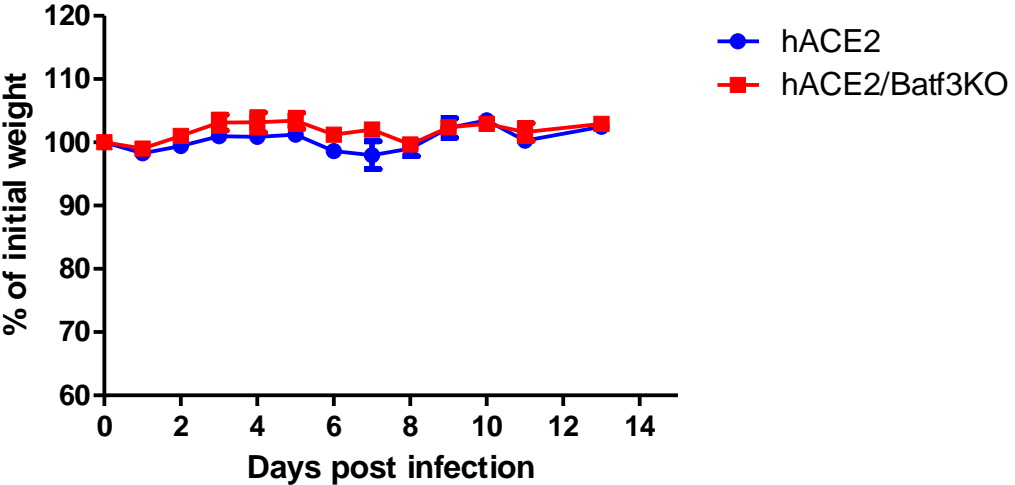

Supplement: S3 Fig — (PDF) [file pone.0294176.s003.pdf]

S4 Fig

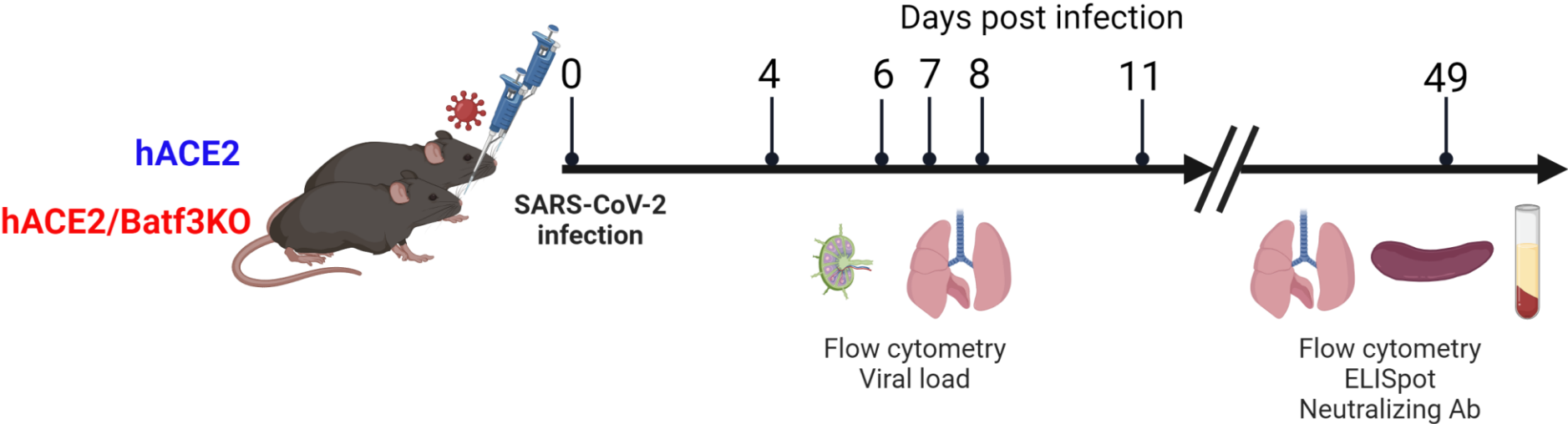

Supplement: S4 Fig — hACE2/Batf3KO (red) and hACE2 (blue) mice were infected with SARS-CoV-2 by i.n. instillation. Mediastinal lymph nodes and Lungs were collected at days 4, 6, 7, 8 and 11 post infection for further analyses (indicated). 7 weeks post infection spleens, lungs and blood were collected for memory immune response evaluation. Graphical illustration was made with Biorender.com. (PDF) [file pone.0294176.s004.pdf]

S5 Fig

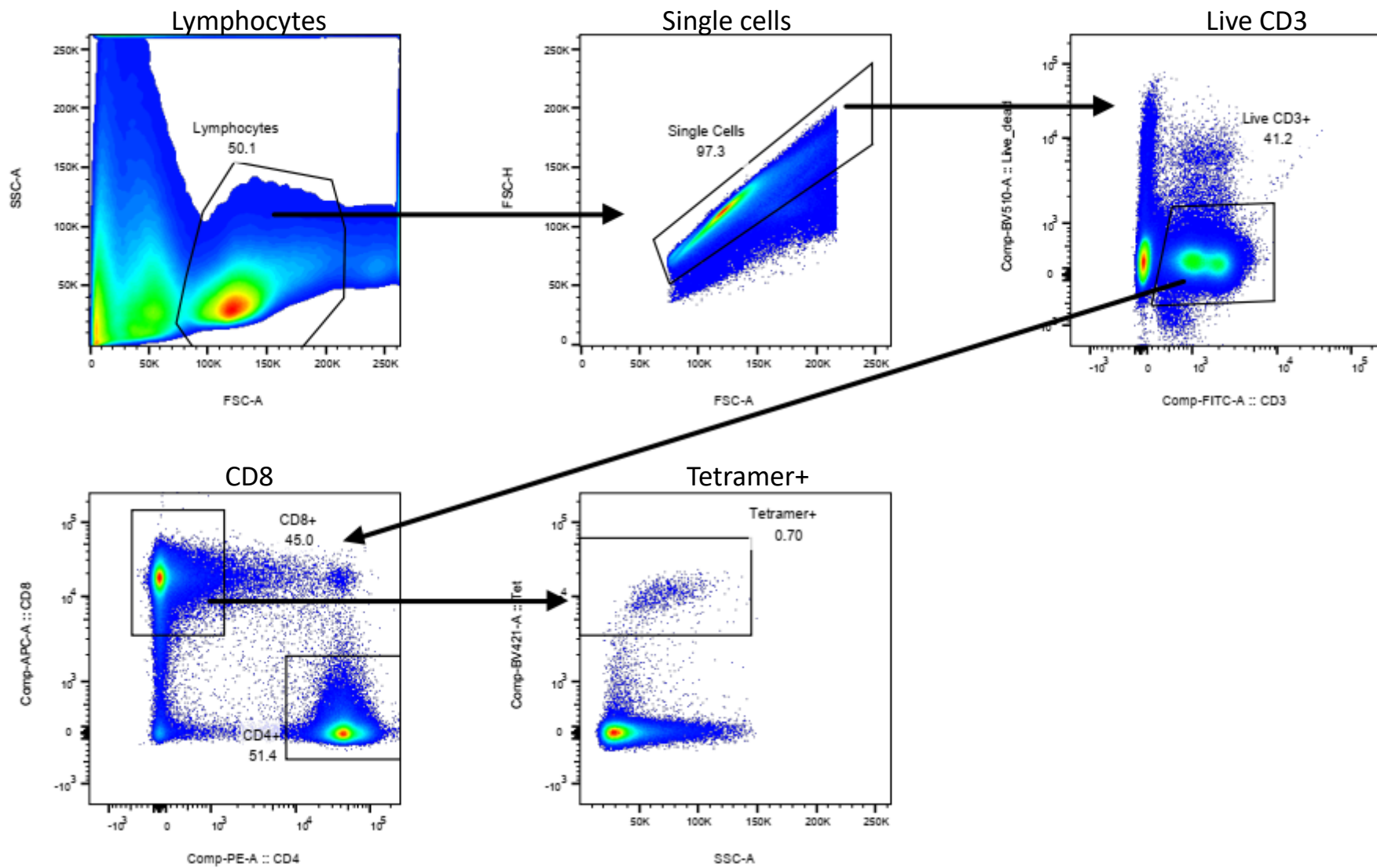

Supplement: S5 Fig — (PDF) [file pone.0294176.s005.pdf]

S6 Fig

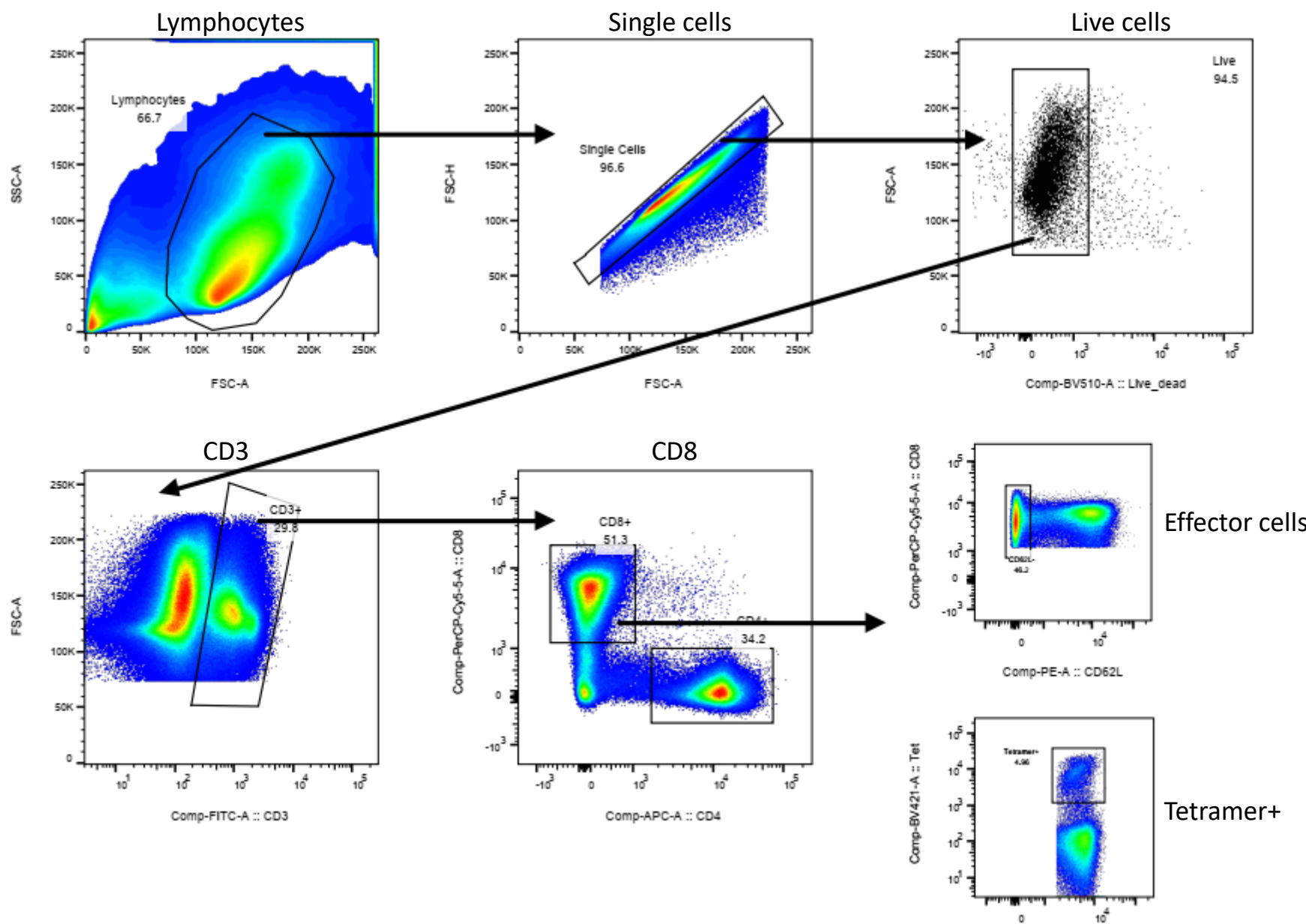

Supplement: S6 Fig — (PDF) [file pone.0294176.s006.pdf]

S7 Fig

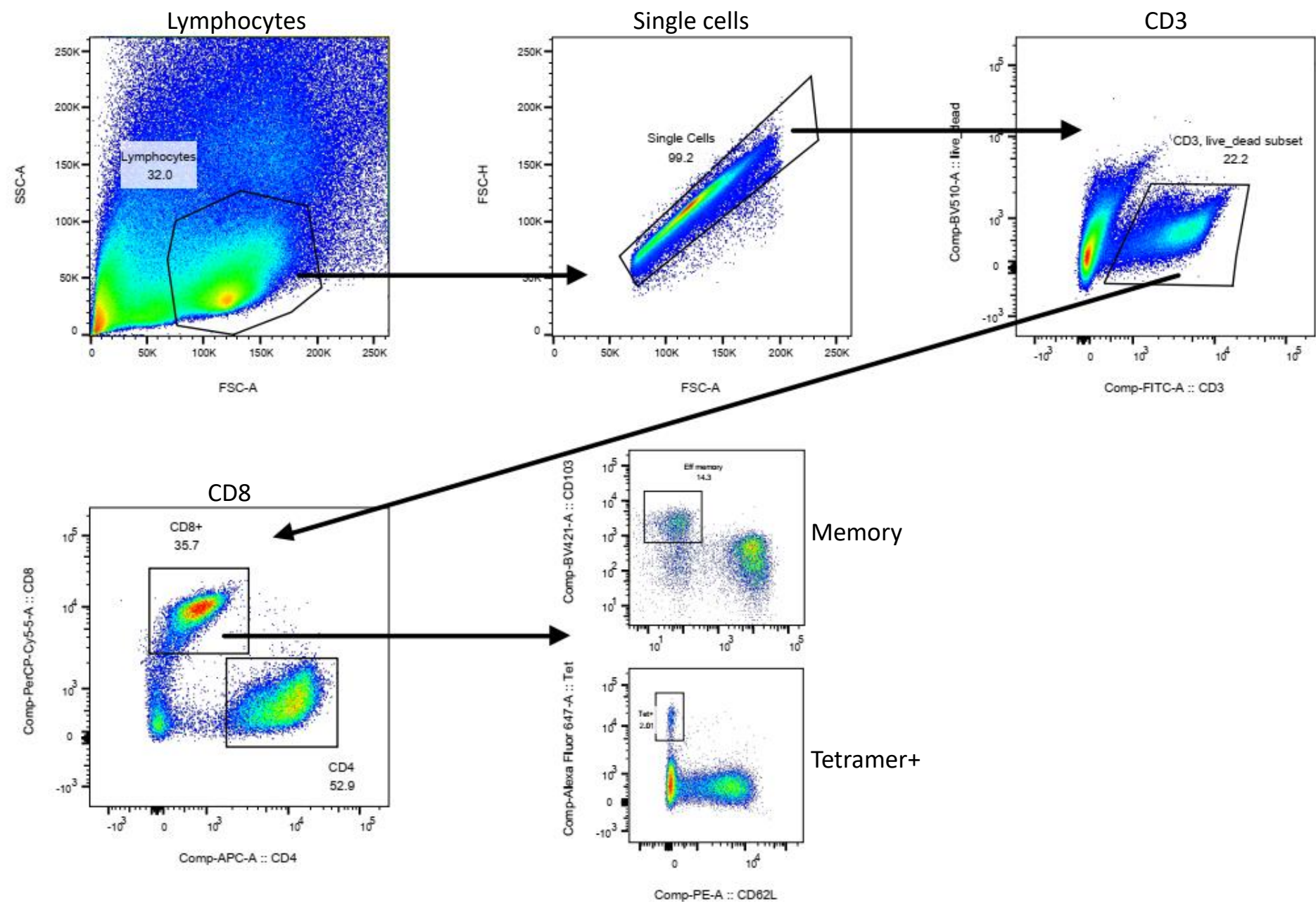

Supplement: S7 Fig — (PDF) [file pone.0294176.s007.pdf]

S8 Fig

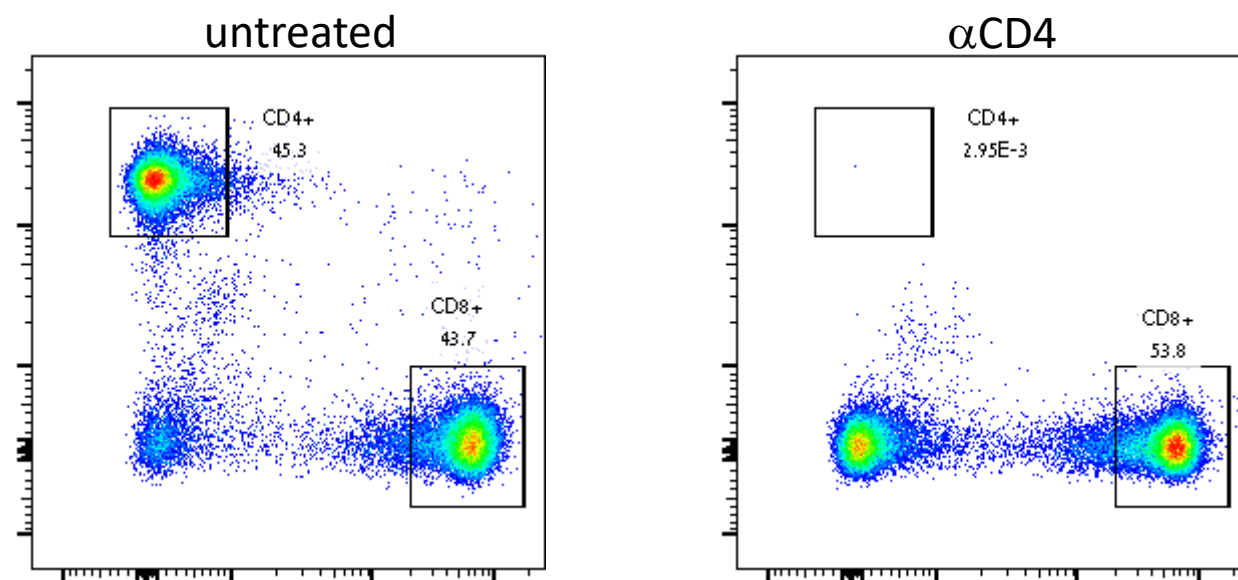

Supplement: S8 Fig — Animals were treated with anti CD4 depleting antibody one day before infection and in every other day post infection. CD4 T-cell depletion was confirmed by flow cytometry. (PDF) [file pone.0294176.s008.pdf]
